# Microbiology of Pressure Ulcers: A Systematic Review

**DOI:** 10.7759/cureus.95912

**Published:** 2025-11-01

**Authors:** Dmitry Shelepenko, Beatriz Jardim, Luís M Almeida, José Miguel Azevedo, Inês Catalão, Miguel Sítima, Gonçalo Tomé

**Affiliations:** 1 Department of Plastic and Reconstructive Surgery and Burns Unit, Coimbra Local Health Unit, Coimbra, PRT; 2 Department of Public Health, Local Health Unit of the Leiria Region, Leiria, PRT; 3 Department of Plastic and Reconstructive Surgery, CUF Santarém, Santarém, PRT

**Keywords:** antibiotic therapy, chronic wounds, microbiology, pressure ulcers, wound infection

## Abstract

Pressure ulcers are complex wounds frequently complicated by infection. This review aims to characterize the distribution of microorganisms in pressure ulcers and secondarily to assess heterogeneity across studies and compare yields between invasive (biopsies) and noninvasive (swabs/irrigation-aspiration) sampling techniques with implications for antimicrobial management. PubMed, Embase, and the Cochrane Library were searched up to 1 January 2025. Original research articles reporting microbiological data from pressure ulcers were included. Studies were excluded if data were incomplete, combined with other wound types, or derived from non-ulcer sampling sites. Risk of bias was assessed using Risk of Bias in Non-randomized Studies - of Interventions (ROBINS-I) V2, ROBINS-E, and RoB 2 frameworks. Data were synthesized descriptively and statistically.

Twenty-eight studies (1,473 patients, 1,682 pressure ulcers, and 4,231 microbial isolates) were included. The most frequently isolated microorganisms were *Staphylococcus aureus *(39.7% of ulcers), *Corynebacterium* spp. (28.8%), *Escherichia coli* (23.1%), *Proteus* spp. (23.1%), *Streptococcus* spp. (22.7%), coagulase-negative *Staphylococcus *(21.6%), *Enterococcus* spp. (20.7%), *Pseudomonas* spp. (17.8%), and *Bacteroides* spp. (16.0%). On average, 2.5 isolates were reported per ulcer (range: 0.5-4.8). Significant heterogeneity was observed across studies for nearly all major organisms (global heterogeneity: χ² = 1958.4, df = 405, p < 0.001). Swabs more frequently yielded *Providencia* spp., *Pseudomonas* spp., *Proteus* spp., and *Enterobacter* spp., whereas biopsies more frequently yielded *Streptococcus* spp., *Enterococcus* spp., and *Klebsiella* spp.

Comparability was limited by the inclusion of diverse populations treated across 14 different countries over the past 50 years. Methodological inconsistencies in sampling and reporting (particularly regarding contaminants, duplicates, and anaerobes) further complicated interpretation and underscore the need for standardized protocols. Nevertheless, findings consistently confirmed a polymicrobial flora dominated by a small group of recurrent species. The ulcer microenvironment is dynamic and distinct from culture media, complicating both diagnosis and treatment. Despite marked heterogeneity, the consistent predominance of *Staphylococcus aureus*, *Enterobacteriaceae*, *Streptococcus* spp., *Enterococcus* spp., *Pseudomonas* spp., and *Bacteroides* spp. provides a valuable framework for empiric therapy. The potential benefits of short perioperative courses of broad-spectrum antibiotics targeting these pathogens warrant further investigation, and prospective controlled trials are urgently needed.

The study protocol was registered in PROSPERO (CRD420251076473) and is available at https://www.crd.york.ac.uk/PROSPERO/view/CRD420251076473.

## Introduction and background

Pressure ulcers, also known as pressure sores, represent a significant clinical challenge with serious implications for both patient outcomes and healthcare systems worldwide [1]. Recent data indicate a prevalence of approximately 12% among hospitalized patients, with even higher rates observed in populations with prolonged immobility, advanced age, or multiple comorbidities [2,3]. The development of pressure ulcers contributes to increased morbidity in this already fragile population, often leading to prolonged hospitalization, increased risk of systemic infection, reduced quality of life, and greater dependency on long-term care [1].

Managing pressure ulcer infection is particularly complex. Differentiating true infection from colonization remains a persistent diagnostic dilemma [4-6], further complicated by the absence of reliable biomarkers or universally accepted criteria [7]. This uncertainty impacts antibiotic decision-making, as there are no strong recommendations for prophylactic use [7,8], and outcomes with empirical treatment remain inconsistent [1,9,10]. Infections are frequently polymicrobial [4,5,11,12], and conventional microbiological testing may yield only a partial view of the microbial landscape, potentially missing clinically relevant pathogens [13].

Surgical management typically involves aggressive debridement of necrotic and infected tissue, elimination of dead space, and appropriate soft tissue coverage to promote healing [1,14]. The presence of underlying osteomyelitis adds further complexity to the treatment plan and may require additional interventions [15,16]. In cases where antibiotic therapy is indicated, treatment is typically guided by microbiological findings [1,4,8], despite the known limitations of culture-based diagnostics [9,13]. Consequently, infection control is frequently suboptimal, and postoperative complications such as wound dehiscence and reinfection remain prevalent [9,10].

This systematic review aims to synthesize the current evidence on the microbiology of pressure ulcers and its implications for clinical management, with a particular focus on patterns of microbial isolates, methodological variability, and a critical discussion of antimicrobial strategies in both surgical and nonsurgical contexts.

## Review

Methods

Protocol and Registration

This systematic review follows the Preferred Reporting Items for Systematic Reviews and Meta-analysis (PRISMA) guidelines [17]. The study protocol was registered on the international register of systematic reviews (PROSPERO) [18] and is available online at https://www.crd.york.ac.uk/PROSPERO/view/CRD420251076473.

Eligibility Criteria

Studies were eligible if they were original research investigating the microbiological profile of pressure ulcers using qualitative microbiological testing, designed to identify and describe the species composition of ulcer flora rather than to quantify the overall bacterial load (which is typically assessed using quantitative methods). Single case reports and conference abstracts or congress presentations were excluded. No language restrictions were applied; however, articles were required to have an English-language abstract for screening purposes. Eligible studies were required to report microbiological data specific to pressure ulcers, including the exact number of microbial isolates, the number of patients, and the pressure ulcers that were sampled. All types of sampling methods, transport media, and identification techniques were accepted, and no restrictions were placed on the type of tissue sampled, provided the microbiological results were clearly attributable to pressure ulcers.

Studies were excluded if they lacked precise reporting on the number of patients with pressure ulcers that were studied and did not mention the number of pressure ulcers sampled; microbiological data were presented in aggregate, selective, or incomplete form (e.g., if only predominant or multiresistant microorganisms were listed); pressure ulcers were grouped with other wound types (e.g., burns and diabetic ulcers) without separately reported microbiological data; samples were taken in peri-lesional skin or other parts of the body (not the pressure ulcer itself). Only studies involving human subjects were eligible.

Information Sources and Search Strategy

A systematic literature search was conducted in PubMed, the Cochrane Library, and Embase to identify studies addressing microbiological aspects of pressure ulcers. The database search covered all articles published up to January 1, 2025, and included controlled vocabulary terms (MeSH and Emtree) along with relevant keywords related to infection, colonization, osteomyelitis, and antimicrobial therapy. In addition to database searches, a manual search was performed in January 2025, comprising both website-based searches and citation tracking from the reference lists of relevant articles and systematic reviews to identify additional eligible studies not captured in the initial search. Full search strategies for each database are provided in Table 1.

**Table 1:** Database search strategy and results Searches were conducted in PubMed, Embase, and the Cochrane Library in January 2025. The number of records shown reflects the total results retrieved at that time before deduplication. Date limits were applied solely for standardization and reproducibility, as all articles available up to the month of the search were included. Publication-type filters were applied to exclude case reports, editorials, guidelines, reviews, and other non-original research articles.

Database	Search strategy
PubMed (460 records)	("Pressure Ulcer"[MeSH Terms] AND ("pressure ulcer/microbiology"[MeSH Terms] OR "Wound Infection"[MeSH Terms] OR "Anti-Infective Agents"[MeSH Terms] OR "Osteomyelitis"[MeSH Terms]) AND ("1900/01/01"[Date - Publication] : "2024/12/31"[Date - Publication]) AND "humans"[MeSH Terms]) NOT ("case reports"[Publication Type] OR "Review"[Publication Type] OR "systematic review"[Publication Type] OR "Meta-Analysis"[Publication Type] OR "scoping review"[Publication Type] OR "network meta analysis"[Publication Type] OR "Guideline"[Publication Type] OR "practice guideline"[Publication Type] OR "Editorial"[Publication Type] OR "Letter"[Publication Type] OR "Comment"[Publication Type] OR "News"[Publication Type] OR "Congress"[Publication Type] OR "Interview"[Publication Type] OR "Lecture"[Publication Type] OR "personal narrative"[Publication Type] OR "Preprint"[Publication Type] OR "expression of concern"[Publication Type] OR "retracted publication"[Publication Type] OR "retraction of publication"[Publication Type] OR "consensus development conference"[Publication Type])
Embase (289 records)	(((pressure OR decubitus OR bed) NEXT/2 (ulcer* OR sore* OR injur*)):ab,ti) AND ('decubitus'/exp/mj AND 'microorganism'/de OR ('decubitus'/exp/mj AND 'wound infection'/exp) OR ('decubitus'/exp/mj AND 'antiinfective agent'/exp) OR ('decubitus'/exp/mj AND 'osteomyelitis'/exp)) AND ('clinical article'/de OR 'clinical protocol'/de OR 'clinical trial'/de OR 'cohort analysis'/de OR 'comparative study'/de OR 'controlled clinical trial'/de OR 'controlled study'/de OR 'in vitro study'/de OR 'major clinical study'/de OR 'medical record review'/de OR 'observational study'/de OR 'prospective study'/de OR 'randomized controlled trial'/de OR 'randomized controlled trial topic'/de OR 'retrospective study'/de) AND 'human'/de NOT ('case report'/de OR 'conference abstract'/it OR 'note'/it OR 'letter'/it OR 'editorial'/it OR 'guideline'/it OR 'practice guideline'/de OR 'review'/it OR 'meta analysis'/it OR 'systematic review'/it) AND [embase]/lim Publication year limits applied: 1900–2024
Cochrane - Clinical trials (368 records)	#1 MeSH descriptor: [Pressure Ulcer] explode all trees and with qualifier(s): [microbiology - MI] – (12 trials) #2 ("Pressure ulcers" OR "Bedsores" OR "Pressure Injuries") AND ("Infection" OR "Colonization" OR "Osteomyelitis" OR "Microorganism" OR "Microbiological") (Word variations have been searched) – (356 trials)

Study Selection

Three reviewers (D.S., G.T., and B.J.) independently screened the titles and abstracts of all identified articles. Abstracts selected by at least one reviewer were retrieved in full text and independently assessed against the pre-established eligibility criteria by two reviewers (D.S. and B.J.). Any disagreements during the full-text selection phase were resolved through discussion and consensus among the reviewers.

Data Collection Process and Data Items

Two reviewers (D.S. and B.J.) independently extracted data from studies meeting final inclusion criteria into a prespecified data collection form. All articles were fully analyzed for the following variables: title; author; publication date; journal; study location, duration, design, and the years during which sample collection took place; number of patients and ulcers studied; patient characteristics including sex and age distribution; ulcer stage and anatomical location; sampling method; culture conditions; presence of osteomyelitis; antibiotic therapy during microbiological sampling; percentage of negative, monomicrobial, and polymicrobial cultures; and the absolute count of each microbiological isolate. If various types of sampling methods were used in the same population and results were presented separately (e.g., studies that compared different sampling techniques), only the most accurate method was considered for further analysis to avoid duplication of results (e.g., tissue biopsy > swabs) [13,19] except in cases where only part of the patients undergo the most accurate sampling method. In interventional comparative studies that assessed microbiological growth in pressure ulcers before and after antiseptic (or other therapy) use, only samples prior to therapy application were considered to avoid bias in microbial isolation. Discrepancies were identified and resolved through discussion.

Risk of Bias Assessment

The methodological quality of the included studies was assessed using the Joanna Briggs Institute (JBI) Levels of Evidence [20-22]. Additionally, risk of bias was assessed using the revised Risk of Bias in Non-randomized Studies - of Interventions (ROBINS-I V2) [23] and the Risk of Bias in Non-randomized Studies - of Exposures (ROBINS-E) [24] frameworks, which evaluate bias across seven domains relevant to non-randomized studies, and the Cochrane Risk of Bias 2 tool (RoB 2) [25], which was applied to assess bias across five domains in three randomized studies. The risk of bias and evidence grading were independently performed by two reviewers (D.S. and G.T.). Any discrepancies between assessments were resolved through discussion.

Synthesis and Statistical Analysis

Isolates were synthesized by microorganism type across studies and summarized as absolute counts and percentages. Tables and graphs were used to illustrate distributional patterns and relative prevalence. Variability in microbial distributions across studies was assessed in two ways: (i) Chi-square tests for heterogeneity of proportions for each microorganism per ulcer count and (ii) Cochran’s Q test with the I² index to quantify heterogeneity in isolate counts. A global Chi-square test of independence was also performed to confirm overall heterogeneity in microbial distributions across studies, with effect size quantified using Cramér’s V. Analyses of anaerobes were restricted to studies that reported them. To compare swab and biopsy sampling methods, Firth penalized logistic regression models were applied, and results were expressed as odds ratios (ORs) with 95% confidence intervals. Statistical significance was defined as p < 0.05. Chi-square and Cochran’s Q/I² analyses were conducted in IBM SPSS Statistics v29.0 (IBM Corp., Armonk, NY) [26] and Firth logistic regression in Python (statsmodels) [27,28]. Formal sensitivity analyses were not performed. However, comparative analyses were conducted: Pooled ORs were calculated to compare microorganism isolation between invasive and noninvasive sampling methods, and subgroup analyses were carried out for studies reporting anaerobic cultures.

Results

Study Selection

**Figure 1:**
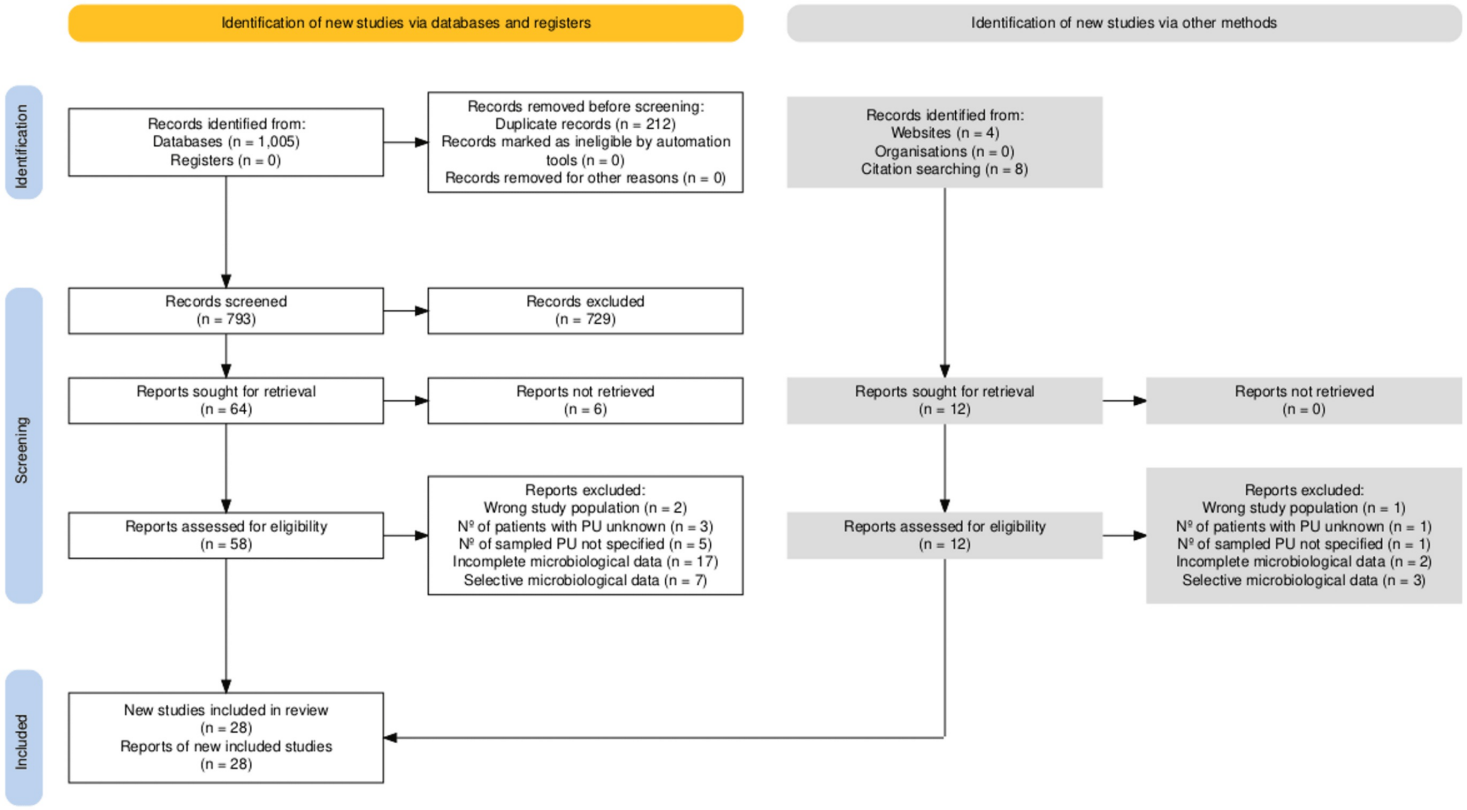
PRISMA flow diagram of the study selection process The diagram summarizes the identification, screening, eligibility, and inclusion of studies in the review. Image credit: Generated using the PRISMA 2020 flow diagram Shiny App (available at https://www.eshackathon.org/software/PRISMA2020.html), © PRISMA Group, CC BY 4.0 [17]. PU = pressure ulcers.

A total of 1005 records were identified through database search (460 through PubMed, 368 through the Cochrane Library, and 289 through Embase). Duplicate records were removed manually using Rayyan, a web-based tool for systematic review screening [29]. Following title and abstract screening, 64 articles were selected for full-text review. An additional 12 records were identified through manual searching, including both website-based searches and citation tracking from reference lists of relevant publications. Of these, 76 (64+12) records were sought for retrieval, and six full texts were unavailable. Among the 70 reviewed articles, 28 met the eligibility criteria (24 from databases and four from manual search) and were included in the final analysis. Forty-two articles were excluded for the following reasons: three focused on a different study population, four did not report the number of patients with pressure ulcers, six failed to specify the number of pressure ulcers sampled, 19 presented incomplete microbiological data that did not allow completion of the predefined data extraction table, and 10 reported only selective bacteriological findings related to their specific study objectives. The study selection process is illustrated in the PRISMA flow diagram (Figure 1) [17].

Study Characteristics

In total, microbiological data were compiled from 28 studies, covering 1,473 patients, 1,682 pressure ulcers, and yielding 4,231 microbial isolates. All major microbiological groups were represented. Table 2 presents the included studies and their main characteristics.

**Table 2:** Characteristics of the included studies, patients, and pressure ulcers This table presents the main data from the studies included in this review. No. = number, M = male, F = female, Y = years, PU = pressure ulcer, ND = no data, SD = standard deviation, IQR = interquartile range, USA = United States of America, UK = United Kingdom, SCI = spinal cord injury, BMI = body mass index.

Authors and year	Title	Site, city, country	Journal	Study design	Sampling period (Years)	No. of patients with sampled PUs	No. of sampled PUs	No. of samples	Sex	Age	Patients' characteristics	Pressure ulcer location	Sampling method	Type of culture
Galpin et al., 1976 [14]	Sepsis associated with decubitus ulcers	Harbor General Hospital, Los Angeles, California, USA	The American Journal of Medicine	Retrospective case series	1970–1974	21	47	ND	14M, 7F	Mean 50.5Y ± 23,8 SD	Patients with sepsis attributed to PUs	Sacrum 12, buttocks 7, trochanter 7, ischium 5, heels 4, knees 3, ankles 3, pretibial 2, finger 1, scalp 1, coccyx 1, malleoli 1	Needle aspiration, surgical drainage, or cotton swab	Aerobic + Anaerobic
Daltrey et al., 1981 [30]	Investigation into the microbial flora of healing and nonhealing decubitus ulcers	School of Health & Applied Sciences, Leeds Polytechnic, Leeds, UK	Journal of Clinical Pathology	Prospective case series	ND	53	74	ND	11M, 42F	Mean 79Y, range 64-97Y	Patients with PUs admitted to the geriatric unit of a large general hospital	ND	Sterile swab	Aerobic
Sugarman et al., 1983 [31]	Osteomyelitis beneath pressure sores	Houston Veterans Administration Medical Center, Huston, Texas, USA	Archives of Internal Medicine	Prospective case series	ND	22	28	28	22M, 0F	ND	Hospitalized patients with PUs stage III or IV that did not heal for at least 2 weeks of local care	Trochanter 12, sacrum 8, femur 2, calcaneus 2, ischium 2, foot 1, amputation site 1	Sterile swab	Aerobic + Anaerobic
Mathes et al., 1983 [32]	Coverage of the infected wound	Department of Surgery, University of California, San Francisco, California, USA	Annals of Surgery	Retrospective case series	1978–1982	17	17	ND	ND	ND	Consecutive patients with chronic infected stage IV PUs that received surgical treatment	Ischium 8, sacrum 7, trochanter 2	Intraoperative samples	ND
Sapico et al., 1986 [5]	Quantitative microbiology of pressure sores in different stages of healing	Rancho Los Amigos Medical Center, Downey, California, USA	Diagnostic Microbiology and Infectious Disease	Prospective cohort study	ND	20	20	ND	ND	ND	Patients with SCI and PUs	Sacrum 19, trochanter 4, ischium 2	Tissue biopsy (of the most necrotic/infected area)	Aerobic + Anaerobic
Ehrenkranz et al., 1990 [33]	Irrigation-aspiration for culturing draining decubitus ulcers: correlation of bacteriological findings with a clinical inflammatory scoring index	Nursing homes in the Miami Area, Florida, USA	Journal of Clinical Microbiology	Prospective cross-sectional diagnostic accuracy study	ND	32	32	64	9M, 23F	Mean 82Y ± 5.8 SD	Nursing home residents with draining PUs stages II (9), III (16), and IV (7)	Sacrum 12, hip 11, foot 9	Sterile swab (irrigation-aspiration technique)	Aerobic + Anaerobic
Brook et al., 1991 [34]	Microbiological studies of decubitus ulcers in children	Fairview State Hospital, Costa Mesa, California, USA; Children’s Hospital, Washington DC, USA	Journal of Pediatric Surgery	Retrospective cross-sectional study	1976–1981	58	58	58	31M, 27F	Mean 6.4Y, range 5-16Y	Hospitalized children with PUs (mental retardation (31), congenital hydrocephalus (12), brain damage (11), hospitalized for surgical conditions (4))	Scalp 18, hands 15, legs 13, buttocks 9, other 3	Curettage or aspiration	Aerobic + Anaerobic
Baum et al., 2001 [35]	Tissue and serum concentrations of levofloxacin in orthopaedic patients	Department of Orthopaedic Surgery, Heidelberg University Hospital, Heidelberg, Germany	International Journal of Antimicrobial Agents	Prospective case series	1999	12	12	48	8M, 4F	Mean 60Y, range 27-78Y	Patients with SCI and PUs that underwent surgical excision and reconstruction with a myocutaneous flap	Ischium 6, trochanter 5, sacrum 1,	Tissue biopsy	Aerobic only
Sopata et al., 2002 [36]	Effect of bacteriological status on pressure ulcer healing in patients with advanced cancer	Department of Palliative Medicine, University of Medical Sciences, Poznań, Poland	Journal of Wound Care	Prospective randomized controlled trial	1996–1999	34	38	38	16M, 18F	Mean 58.6Y, range 24-88Y	Patients with advanced cancer and PUs stage II (12) and III (26)	Sacrum, buttocks, coccyx, lower limbs, other	Sterile swab	Aerobic + Anaerobic
Sipponen et al., 2008 [37]	Beneficial effect of resin salve in treatment of severe pressure ulcers: a prospective, randomized and controlled multicenter trial	11 primary care hospitals across Finland	British Journal of Dermatology	Prospective randomized controlled trial	2005–2007	22	29	ND	9M, 13F	Mean 77.5Y, range 58-98Y	Hospitalized patients with PUs stage II (12), stage III (14), and stage IV (3), considered not suitable for surgical treatment, with life expectancy > 6 months and without malignant disease	Calcaneus 10, ischium 6, trochanter 4, sacrum 3, other 6	Not specified	Aerobic + Anaerobic
Serena et al., 2009 [38]	The impact of noncontact, nonthermal, low-frequency ultrasound on bacterial counts in experimental and chronic wounds	3 wound care facilities located in northwestern Ohio; Erie, Pennsylvania; and Chicago, Illinois, USA	Wounds	Prospective quasi-experimental study	2006–2007	11	11	11	6M, 5F	Mean 60Y, range 44-96Y	Consecutive, adult in- and outpatients with stage III PUs and wound volume <160 cm^3^, with no clinical signs of acute wound infection and who completed both pre- and post-treatment biopsies	Ischium 6, sacrum 3, hip 1, great toe 1	Tissue biopsy	ND. Only aerobic results were reported
Ghaly et al., 2010 [39]	Control of bacterial contamination of bed sores by using some natural extracts	El-Wafaa & El-Amel Hospital, Cairo, Egypt	Journal of Applied Sciences Research	Cross-sectional (clinical isolates) + lab experimental arm	2007	35	35	35	21M, 14F	Range 35-75Y	Patients with PUs	ND	Sterile swab	ND. Only aerobic results were reported
Larson et al., 2012 [40]	Protocol management of late-stage pressure ulcers: a 5-year retrospective study of 101 consecutive patients with 179 ulcers	Froedtert Hospital, Medical College of Wisconsin, Milwaukee, Wisconsin, USA	Plastic and Reconstructive Surgery	Retrospective cohort study	2004–2009	68	121	121	ND	ND	Consecutive patients with SCI and PUs who had surgery and immediate reconstruction	Ischium, sacrum, trochanter, and other sites	Bone biopsy	ND
Biglari et al., 2012 [41]	Use of Midihoney as a nonsurgical therapy for chronic pressure ulcers in patients with spinal cord injury	Department of paraplegiologie of BG Trauma Center, Ludwigshafen, Germany	Spinal Cord	Prospective case series	2008	20	20	ND	13M, 7F	Mean 48.7Y, range 30-79Y	Patients with SCI and PUs stages III and IV, who had previously undergone unsuccessful dressings treatment for a minimum of 12 weeks	Sacrum 9, leg 4, ischium 3, heel 2, groin 1, thigh 1	Sterile swab	Aerobic only
Hossain et al., 2012 [42]	Bacteriological status of pressure sore - a study of 50 cases	Department of Plastic Surgery, Dhaka Medical College Hospital, Dhaka, Bangladesh	Bangladesh Journal of Plastic Surgery	Prospective case series	2009–2011	50	74	74	36M, 14F	Mean 47.4Y ± 13.3 SD	Patients admitted to plastic surgery department with PUs stages III (36) and IV (29), other (9)	Sacrum 42, trochanter 27, ischium 2, iliac crest 2, heel 1	Sterile swab (Gloucestershire protocols)	ND
Mizokami et al., 2014 [43]	Necrotizing soft tissue infections developing from pressure ulcers	National Center for Geriatrics and Gerontology (NCGG Hospital), Obu, Aichi Prefecture, Japan	Journal of Tissue Viability	Retrospective case series	2005–2012	24	24	24	10M, 14F	Mean 82.9Y ± 9.2 SD	Hospitalized patients with necrotizing soft tissue infections that developed from PUs	Sacrum 11, trochanter 4, coccyx 4, ischium 2, foot 1, ilium 1, shoulder 1	Deep tissue biopsy	ND
Ciliberti et al., 2014 [44]	Effective management of pressure ulcers using hydrofiber technology with silver ions	Microbiology Unit of San Leonardo Hospital in Gragnano, Naples, Italy	Wound Medicine	Prospective case series	2012	20	20	20	ND	ND	Adult patients with PUs stages III and IV without eschar or necrosis, treated at home-care setting in Napoli South area	Sacrum 10, trochanter 6, leg 2, foot 1, occipital 1	Tissue biopsy	Aerobic only
Brenta et al., 2014 [45]	Infections and pressure sores	Plastic and Reconstructive Surgery Unit of the University of Pavia, Salvatore Maugeri Research and Care Institute, Pavia, Italy	Acta Vulnologica	Retrospective case series	2011–2013	56	69	126	ND	ND	Patients with PUs treated in a plastic surgery unit in whom bacterial culture was performed	ND	Sterile swab (irrigation-aspiration technique)	Aerobic + anaerobic but only aerobic results were reported
Brunel et al., 2015 [46]	Diagnosing pelvic osteomyelitis beneath pressure ulcers in spinal cord: a prospective study	Montpellier University Hospital, Montpellier, France	Clinical Microbiology and Infection	Prospective cross-sectional diagnostic accuracy study	2011–2014	28	35	122	22M, 6F	Median 51Y	SCI patients with diagnosed or suspected osteomyelitis with pelvic PUs stages III (5) and IV (39) with unfavorable evolution despite optimal treatment and indication for hospitalization for surgical debridement	Ischium 24, sacrum 15, trochanter 5	Bone biopsy	Aerobic + Anaerobic
Balbuena et al., 2015 [47]	Microbiology of pressure and vascular ulcer infections	Chronic Wounds Unit, Puerta de Hierro Majadahonda University Hospital, Madrid, Spain	Revista Española de Geriatría y Gerontología	Prospective cross-sectional study	2011–2012	69	69	69	32M, 37F	Median 83Y, 45-97 IQR	Outpatients with infected PUs (excluding traumatic, oncologic, neuropathic and post-op cases) stages II (4), III (17) and IV (48)	Heels 25, sacrum 22, leg 8, trochanter 6, ischium 5, foot 3	Sterile swab	Aerobic + Anaerobic
Kamradt et al., 2017 [48]	Bacterial load of conditioned pressure ulcers is not a predictor for early flap failure in spinal cord injury	Heidelberg University Hospital, Heidelberg, Germany	Spinal Cord	Prospective cohort study	2012–2013	40	40	40	31M; 9F	ND	Consecutive SCI patients with PUs stages III and IV admitted to surgical repair	ND	Tissue biopsy	Aerobic + anaerobic, but anaerobic results were not differentiated
Tedeschi et al., 2017 [9]	Superficial swab versus deep tissue biopsy for the microbiological diagnosis of local infection in advanced-stage pressure ulcers of spinal-cord-injured patients: a prospective study	Specialized care unit dedicated to SCI patients with pressure ulcers, Montecatone Rehabilitation Institute, Imola, Italy	Clinical Microbiology and Infection	Prospective cross-sectional diagnostic accuracy study	2011–2014	116	116	ND	103M, 13F	Median 49Y, 39-63 IQR	SCI patients with PUs stages III (9), IV (98) and unstageable (9) undergoing scheduled surgical debridement and reconstruction	Ischium 60, sacrum 28 and trochanter 13, and other sites 15	Bone and soft tissue biopsy	Aerobic + Anaerobic
Carabelli et al., 2018 [49]	Treatment of pressure ulcer-related pelvic osteomyelitis	Orthopedic Trauma Section, Hospital Italiano de Buenos Aires, Argentina	Revista de la Asociación Argentina de Ortopedia y Traumatología	Retrospective case series	2010–2017	15	27	ND	13M, 2F	Mean 44.9Y, range 22-81Y	Patients with chronic osteomyelitis associated with stage IV pelvic PUs that were subject to surgery	Ischium 13, sacrum 9, and trochanter 5	Bone and soft tissue biopsy	ND. Only aerobic results were reported
Brauncajs et al., 2018 [50]	Impact of low-level laser therapy on the dynamics of pressure ulcer-induced changes considering an infectious agent and cathelicidin LL-37 concentration: a preliminary study	Internal Medicine Ward of Pabianice Medical Centre, Pabianice, Poland	Advances in Dermatology and Allergology	Prospective quasi-experimental study	ND	6	6	ND	3M, 3F	Mean 77.3Y ± 7.5 SD	Hospitalized patients with PUs stage II and III subjected to laser therapy treatment by means of BTL-4000 with laser shower	ND	Sterile swab	Aerobic only
Vasconcelos et al., 2022 [51]	Microbiological identification and resistance profile of microorganisms in pressure injuries after the use of polyhexamethylene biguanide: a series of fourteen cases	Hospital Universitário Pedro Ernesto, Rio de Janeiro, Brazil	Wounds	Prospective quasi-experimental study	2019–2020	14	14	14	10M, 4F	Mean 54.5Y ± 15.2 SD	Hospital admitted patients with PUs stages III (9) and IV (5)	Sacrum 9, malleoli 2, calcaneus 2, and trochanter 1	Sterile swab (Levine technique)	Aerobic only
Rigazzi et al., 2022 [52]	Osteomyelitis and antibiotic treatment in patients with stage IV pressure injury and spinal cord lesion	Swiss Paraplegic Centre, Nottwil, Switzerland	Spinal Cord	Retrospective cohort study	2010–2015	117	125	321	87M, 30F	Median 53Y, 22.3 IQR width	Consecutive SCI patients with stage IV PUs admitted for reconstructive surgical procedures	Ischium 75, sacrum 32, trochanter 10	Bone biopsy	Aerobic + Anaerobic
Dinh et al., 2023 [53]	Short antibiotic treatment duration for osteomyelitis complicating pressure ulcers: a quasi-experimental study	University Hospital Raymond-Poincaré, Garches, France	Open Forum Infectious Diseases	Retrospective quasi-experimental study	2016–2020	415	441	ND	322M, 93F	Mean 53Y ± 14.4 SD	SCI patients with presumed osteomyelitis complicating perineal PUs who underwent surgery and had significant microbiological identification on intraoperative samples	Ischium 267, sacrum 134, trochanter 40	Bone and soft tissue biopsy	Aerobic + anaerobic, but anaerobic results were not differentiated
Garg et al., 2025 [54]	Evaluating the effect of BMIs on wound complications after the surgical closure of pressure injuries	Northwestern Memorial Hospital, Chicago, Illinois, USA	Surgeries	Prospective randomized controlled trial	2016–2019	80	80	ND	53M, 27F	Mean 48,5Y	Adult patients with PUs of different etiologies, stages III and IV admitted for surgical closure of their pressure injury	ND	Tissue biopsy	ND. Only aerobic results were reported

Risk of Bias and Evidence Level of Studies

Most of the included studies were observational in design and corresponded to level 3 or 4 evidence according to the JBI classification [20-22] (cohort studies, analytic or descriptive case series, and diagnostic yield studies). Several diagnostic accuracy studies were identified (Level 2.b), while only three randomized controlled trials were available (Level 1.c). A small number of quasi-experimental studies (Level 2.c-2.d) were also included.

**Figure 2:**
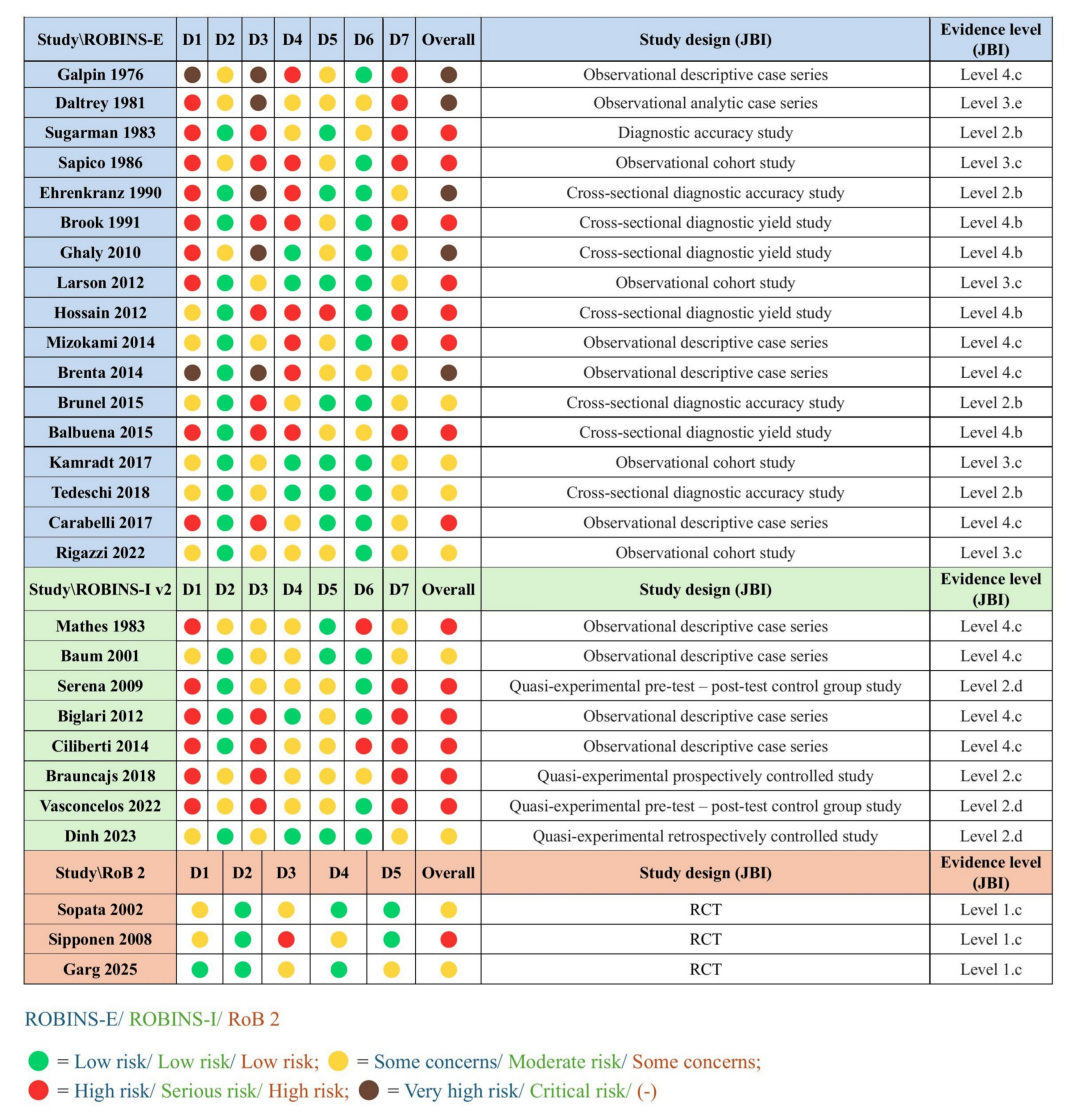
Study design, evidence level, and risk of bias domains Assessed using JBI levels of evidence, ROBINS-E, and ROBINS-I V2 frameworks for nonrandomized studies, covering seven bias domains, and RoB 2 framework for randomized trials, covering five bias domains. D = domain, RCT = randomized controlled trial, JBI = Joanna Briggs Institute, ROBINS-E = Risk of Bias in Non-randomized Studies - of Exposures, ROBINS-I V2 = Risk of Bias in Non-randomized Studies - of Interventions Version 2, RoB 2 = Cochrane Risk of Bias 2 tool.

The overall risk of bias was rated as high/serious or moderate/some concerns in most studies, with five studies presenting very high overall risk of bias, primarily due to confounding, selection of participants, and selective reporting of results. No study demonstrated consistently low risk across all ROBINS-I, ROBINS-E, and RoB 2 domains [23-25]. Notably, most studies demonstrated a low risk of bias in the assessment of exposures and outcomes, suggesting that these particular limitations are unlikely to substantially affect the overall synthesis of microbiological counts across studies. However, the high risk of bias from confounding, participant selection, and selective reporting limits the ability to generalize findings and highlights the substantial heterogeneity in isolate counts driven by external factors unrelated to the microbiological status of the ulcers themselves. The overall high risk of bias also significantly restricts the reliability of conclusions regarding clinical outcomes or treatment effects, though this was not the primary aim of the present review. A detailed summary of study design, JBI evidence level, and risk of bias assessments (ROBINS-I V2, ROBINS-E, and RoB 2) is presented in Figure 2.

Study Outcomes

When excluding likely contaminants such as *Corynebacterium* spp. (28.8% of ulcers) and coagulase-negative *Staphylococcus* (21.6%) [55], the most frequently isolated pathogens were *Staphylococcus aureus* (39.7% of ulcers), *Escherichia coli* (23.1%), *Proteus *spp. (23.1%), *Streptococcus *spp*. *(22.7%), *Enterococcus *spp. (20.7%), *Pseudomonas *spp. (17.8%), and *Bacteroides *spp. (16.0%). Other notable organisms included *Peptostreptococcus *spp. (9%), *Klebsiella *spp. (8%), *Enterobacter* spp. (4.6%), *Morganella morganii* (4.0%), and *Acinetobacter* spp. (3.9%). *Citrobacter* spp., *Providencia stuartii*,* *and *Candida* spp. were rarely isolated, each identified in less than 2% of pressure ulcers. These overall findings are consistent with those reported in previous reviews [56-60].

Eighteen studies reported the number of negative cultures (no growth), 12 provided detailed monomicrobial findings (single-organism growth), and 14 presented data on polymicrobial results (two or more organisms). In total, only 12 studies presented the complete distribution of negative, monomicrobial, and polymicrobial culture results. The proportion of culture-negative ulcers ranged from 0% to 48%, although most reported values of 0% or below 10%. Among the studies reporting polymicrobial results, proportions varied between 20% and 97%, with 10 studies documenting a clear predominance of polymicrobial cultures. Collectively, these findings indicate that the complete absence of bacterial growth is rare and support the current understanding of pressure ulcers as complex microbial ecosystems hosting a diverse range of microorganisms [4,5,11,12]. 

**Figure 3:**
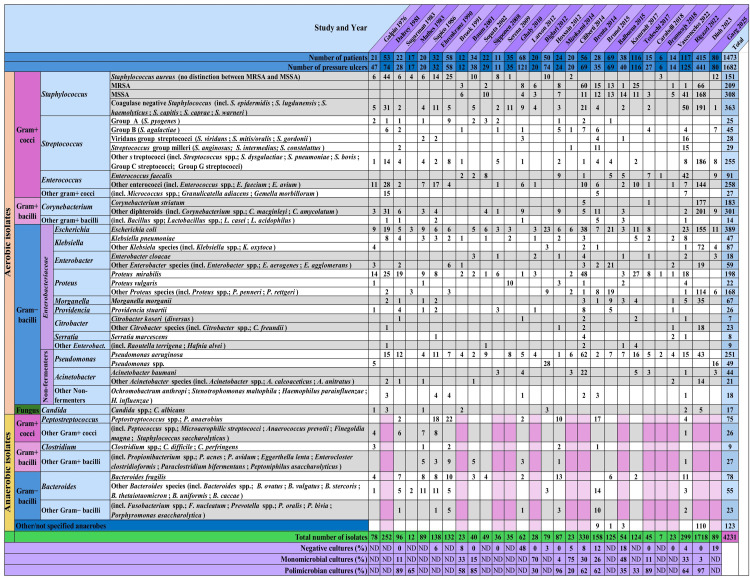
Absolute counts of reported microorganisms in the 28 included studies This table presents the absolute counts of reported microorganisms across all included studies, as well as the proportions of negative, monomicrobial, and polymicrobial cultures. incl = including; ND = no data; spp. = species; gram+ = gram positive; gram− = gram negative; Enterobact. = Enterobacteriaceae; MRSA = methicillin-resistant Staphylococcus aureus, MSSA = methicillin-susceptible Staphylococcus aureus.

**Figure 4:**
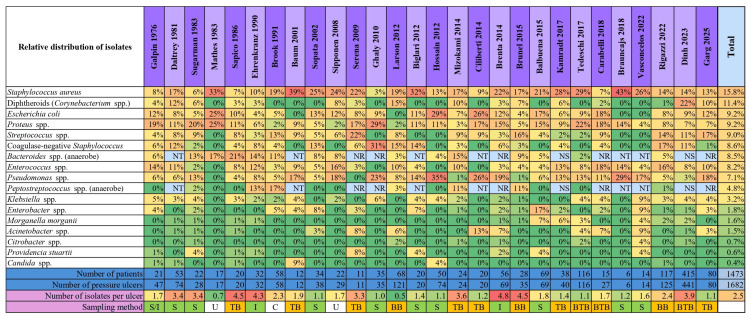
Relative distribution (%) of the 17 most common microbial isolates within each study Values represent the proportion of each microorganism relative to the total number of isolates reported per study (data on Bacteroides spp. and Peptostreptococcus spp. were excluded from studies that did not test (NT), did not report (NR), or did not specify (NS) anaerobic cultures/results). S = swab, I = irrigation-aspiration, TB = tissue biopsy, BB = bone biopsy, BTB = bone and tissue biopsy, U = unknown, C = combined methods (curettage/aspiration), NT = not tested, NR = not reported, NS = not specified, spp. = species.

**Figure 5:**
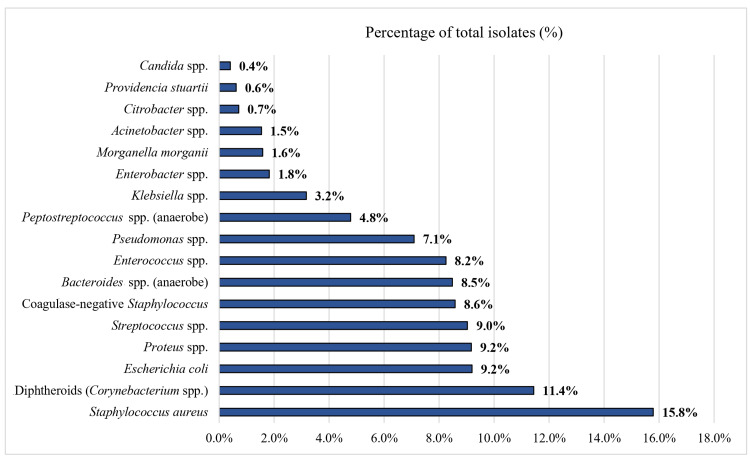
Relative distribution (%) of the 17 most common microbial isolates across all included studies Values calculated relative to the total number of reported isolates (n = 4231 for aerobes, and n=1568 for anaerobes) across all included studies (28 for aerobes and 15 for anaerobes). spp. = species.

The absolute counts of reported microorganisms, as well as the proportions of negative, monomicrobial, and polymicrobial cultures, are shown in Figure 3. The relative distribution of isolates within each study and across all included studies are shown in Figures 4 and 5.

Statistical Assessment of Heterogeneity in Microbiological Distributions

There was statistically significant variability in the distribution of isolates across studies for nearly all major microorganisms (p < 0.05), with the exception of *Klebsiella* spp. and *Citrobacter* spp (Table 3). Nearly half of the studies did not perform anaerobic cultures, failed to report them, or presented results without differentiation and were therefore excluded from the analysis of distribution of isolates for *Bacteroides* spp. and *Peptostreptococcus* spp.. The global Chi-square test of independence (performed on aerobic data only) confirmed substantial heterogeneity in microbial distributions across all studies (χ² = 1958.4, df = 405, p < 0.001), with a small-to-moderate effect size (Cramér’s V = 0.185).

**Table 3:** Microbial isolates and statistical variability across studies A p-value < 0.05 was considered statistically significant. *Variability per ulcer estimated with the Chi-square test for homogeneity of proportions. **Variability per isolate count estimated with Cochran’s Q statistic and I² index. I² values represent the proportion of total variability attributable to true heterogeneity rather than sampling error (25% = low, 50% = moderate, 75% = high). spp. = species; No. = number; PU = pressure ulcers.

Microorganism	No. of studies	Microorganism count	Measure of heterogeneity per ulcer*				Measure of heterogeneity per isolate count**				
			Total no. of sampled PU	Global proportion	χ²	p-value	Total no. of isolates	Global proportion	Q	I²	p-value
*Staphylococcus aureus*	28	668	1682	39.7%	211.2	p < 0.001	4231	15.8%	100.1	73.0%	p < 0.001
Diphtheroids (*Corynebacterium* spp.)	28	484	1682	28.8%	733.4	p < 0.001	4231	11.4%	366.2	92.6%	p < 0.001
*Escherichia coli*	28	389	1682	23.1%	131.1	p < 0.001	4231	9.2%	94.8	71.5%	p < 0.001
*Proteus *spp.	28	388	1682	23.1%	182.1	p < 0.001	4231	9.2%	134.9	80.0%	p < 0.001
*Streptococcus* spp.	28	382	1682	22.7%	247.6	p < 0.001	4231	9.0%	101.5	73.4%	p < 0.001
Coagulase-negative *Staphylococcus*	28	363	1682	21.6%	261.7	p < 0.001	4231	8.6%	148.7	81.8%	p < 0.001
*Enterococcus* spp.	28	349	1682	20.7%	164.8	p < 0.001	4231	8.2%	92	70.7%	p < 0.001
*Pseudomonas* spp.	28	300	1682	17.8%	291.7	p < 0.001	4231	7.1%	321.6	91.6%	p < 0.001
*Klebsiella* spp.	28	134	1682	8.0%	76.8	p < 0.001	4231	3.2%	31.5	14.4%	p = 0.25
*Bacteroides* spp. (anaerobe)	15	133	833	16.0%	210.4	p < 0.001	1568	8.5%	53.3	73.7%	p < 0.001
*Enterobacter* spp.	28	77	1682	4.6%	144.4	p < 0.001	4231	1.8%	208.2	87.0%	p < 0.001
*Peptostreptococcus* spp. (anaerobe)	15	75	833	9.0%	271.5	p < 0.001	1568	4.8%	122.2	88.5%	p < 0.001
*Morganella morganii*	28	67	1682	4.0%	58.8	p < 0.001	4231	1.6%	52.8	48.9%	p = 0.002
*Acinetobacter* spp.	28	65	1682	3.9%	193.3	p < 0.001	4231	1.5%	147	81.6%	p < 0.001
*Citrobacter *spp.	28	30	1682	1.8%	36.1	p = 0.11	4231	0.7%	20.7	0.0%	p = 0.80
*Providencia stuartii*	28	26	1682	1.5%	167.9	p < 0.001	4231	0.6%	128.2	78.9%	p < 0.001
*Candida *spp.	28	17	1682	1.0%	55.9	p < 0.001	4231	0.4%	76.8	64.8%	p < 0.001

Comparison of Microorganism Isolation by Sampling Method

**Figure 6:**
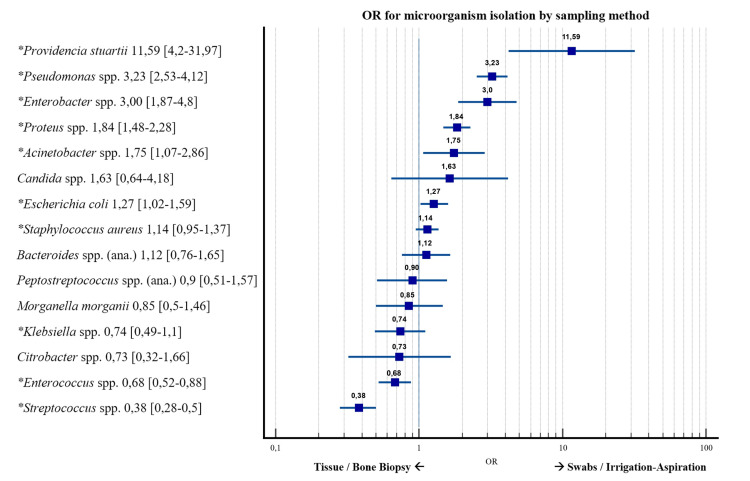
Forest plot of odds ratios (OR), with 95% confidence intervals, comparing invasive (tissue/bone biopsy) and noninvasive (swab or aspiration/irrigation) sampling methods for all major pathogenic microorganisms OR > 1 indicates higher odds of isolation in noninvasive samples, whereas OR < 1 indicates higher odds in invasive samples. Microorganisms marked with an asterisk (*) showed statistically significant differences (p < 0.05). OR = odds ratio, spp. = species.

Firth’s logistic regression was used to compare the relative proportions of microorganisms obtained by invasive (tissue or bone biopsy) and noninvasive (swab or irrigation-aspiration) sampling methods [11,13,58]. Three studies were excluded because the sampling method was either unclear or combined invasive and noninvasive approaches. Overall, 1,231 isolates were obtained from swabs and 2,807 from biopsies, corresponding to 30.5% and 69.5% of all isolates, respectively. When restricting the analysis to studies that reported anaerobes, 556 isolates were recovered from swabs (40.4%) and 819 (59.6%) from biopsies. Details of sampling methods and excluded articles are provided at the bottom of Figure 4. Several microorganisms showed significant differences in their distribution: *Pseudomonas* spp., *Proteus* spp., *Enterobacter* spp., and *Providencia* spp. were more frequently isolated from noninvasive samples, whereas *Streptococcus* spp., *Enterococcus* spp., and *Klebsiella* spp. were more commonly detected in invasive biopsies. Among the studies that reported anaerobic findings, no significant differences were observed for *Bacteroides* spp. or *Peptostreptococcus* spp. A forest plot of ORs, with 95% confidence intervals, illustrating the relative likelihood of isolation by noninvasive versus invasive sampling methods for all pathogenic microorganisms, is shown in Figure 6.

Reporting Biases

The exclusion of studies without an English abstract and reliance on indexed literature may have led to the omission of some relevant reports, particularly among older publications. Although manual searching and citation tracking were performed to mitigate this risk, the possibility of missed studies remains. We also consider the presence of publication bias as possible as studies reporting striking or clinically relevant microbiological findings (such as a high prevalence of resistant organisms) are more likely to be published than those with unremarkable results, which may overestimate or distort the distribution of isolates.

Although we attempted to minimize selective reporting bias by excluding all articles that provided only partial, incomplete, or highly specific bacteriological data, many studies likely removed potential contaminants (notably saprophytic flora) from their bacteriological counts, thereby contributing to heterogeneity in the distribution of isolates. Complete anaerobic data were reported in only 15 of 28 studies, and in two additional studies, anaerobes were cultured but not differentiated to the species level. This was not considered a reason for study exclusion, as many centers do not routinely perform anaerobic cultures due to technical limitations. However, to reduce bias arising from these inconsistencies, the calculation of variability in the distribution of anaerobic isolates across studies and the comparison of sampling methods for anaerobes were restricted to the 15 studies that reported anaerobic counts. For similar reasons, the two studies that did not specify the sampling method, along with the study that used a combined approach, were excluded from the comparative analysis of sampling methods.

No evidence of duplicate publication of isolate data was identified, either between studies and within studies, since only one sampling method was allowed to be included in diagnostic accuracy studies, and only one single time window was considered when ulcers were evaluated at different healing stages or time periods. However, because many studies relied on retrospective data extracted from medical records, it is possible that some ulcers were sampled and reported multiple times. Ideally, each ulcer should have been tested only once, with a consistent number of samples across ulcers, and if the same pathogen was repeatedly isolated from the same ulcer across different samples, it should have been counted only once to avoid duplication. Unfortunately, only five out of 28 studies explicitly reported removing duplicate isolates under these circumstances.

These reporting biases, together with inconsistencies in the reporting of contaminants and anaerobes across studies, likely contributed to the wide range in the number of microorganisms reported per ulcer (average ~ 2.5; range: 0.5-4.8), underscoring the importance of evaluating microbiological findings also in terms of the relative distribution of isolates within each study (see Figure 4 and Figure 2).

Finally, the risk of reviewer bias in this review was considered low. Each article was independently analyzed multiple times by two reviewers (D.S. and B.J.) to ensure that patient, ulcer, and pathogen counts were extracted with maximum accuracy. All microorganism species that were reported by the included studies were entered into our dataset, and aggregation of low-frequency isolates was performed only after data collection and immediately prior to statistical analysis.

Certainty of Evidence

The certainty of the overall body of evidence was limited by the predominance of observational designs, most of which corresponded to Level 3 or 4 evidence according to the JBI classification [20-22] (cohort studies, case series, and diagnostic yield studies). Only a small number of quasi-experimental studies (Level 2.c-2.d), diagnostic accuracy studies (Level 2.b), and randomized controlled trials (Level 1.c) were identified. The certainty of evidence for the distribution of microorganisms (primary outcome) was rated as moderate, since this outcome was consistently reported across studies and supported by a large number of isolates (>4,000). However, differences in sampling methods, microbiological identification techniques, and selective reporting of pathogens (e.g., exclusion of contaminants and inconsistent reporting of anaerobes) introduced heterogeneity, thereby reducing confidence in absolute proportions.

Regarding the heterogeneity of microbiological distribution across studies (secondary outcome), although significant variability was demonstrated statistically, the certainty of evidence was rated as low, as this heterogeneity was largely attributable to methodological differences, such as sampling techniques, culture protocols, reporting practices, and patient characteristics, making it difficult to distinguish true biological diversity from study-related inconsistencies.

For the comparative yield of microorganisms between swab and biopsy sampling (secondary outcome), the certainty of evidence was also rated as low. Although only three of the 28 studies were excluded because the sampling method was combined or not specified, the main limitation arose from the fact that swabs and biopsies were performed in very different populations, reducing the validity of direct comparisons. Moreover, the two groups did not include a similar proportion of isolates, with roughly two-thirds originating from biopsies, whereas ideally both sampling methods would have been applied to the same ulcers within the same population. In addition, all invasive techniques were grouped together, despite substantial differences between procedures (e.g., bone biopsy versus soft tissue biopsy), and the same occurred for noninvasive methods, where simple swabbing, the Levine technique, and irrigation methods were all analyzed as a single category. These methodological inconsistencies, combined with small sample sizes for some organisms and variable reporting practices (duplicate isolates and unreported contaminants), reduced precision, and further lowered the certainty of evidence.

Imprecision could not be formally assessed using confidence intervals, since isolated counts represent absolute values rather than statistical estimates. Nevertheless, variability in sample sizes and reporting practices limited comparability across studies. Despite these limitations, the inclusion of 28 studies and more than 4,000 isolates provides the most comprehensive synthesis of microbiological data from pressure ulcers to date, with an overall certainty of evidence judged to be moderate to low [17,61].

Discussion

Variability in Microbial Distributions Across Studies

Our analysis revealed statistically significant variability in the distribution of most microorganisms across studies (p < 0.05). This heterogeneity appears to stem primarily from methodological inconsistencies rather than true biological differences [13]. While patient characteristics and clinical context may also contribute to these discrepancies, the dominant sources of variation likely include differences in sampling techniques, tissue types, transport conditions, culture methods, and the availability and sensitivity of microbiological identification systems [5,9,19]. The global analysis confirmed this pattern, with a highly significant Chi-square test indicating strong overall heterogeneity (χ² = 1958.4, df = 405, p < 0.001). However, the effect size was only in the small-to-moderate range (Cramér’s V = 0.185), suggesting that although differences are real and systematic, they are not so extreme that each study represents a completely unique microbial profile. In practical terms, this indicates that there is some shared structure across studies, common organisms tend to be reported consistently, yet their relative frequencies vary considerably depending on study design and methodological context [56,59]. Although the absence of a formal sensitivity analysis can be acknowledged as a limitation of this review, it would likely have provided limited additional insight, as the correspondence between the overall risk of bias and the quality of microbiological assessment appeared poor, with more than 70% of studies rated as having a high overall risk of bias.

Microbiological Spectrum of Pressure Ulcers

On average, *Staphylococcus aureus* remained the most frequently isolated microorganism across studies [56,62], although it was not the leading isolate in every individual report. While it is commonly part of the normal skin flora, it is well known for its propensity to colonize wounds of all types, including pressure ulcers [11,63]. The distinction between methicillin-resistant (MRSA) and methicillin-sensitive *Staphylococcus aureus* (MSSA) became standard in the literature around the year 2000 [56,64]. The proportion of MRSA among *S. aureus* isolates varied considerably between studies, ranging from settings where nearly all isolates were resistant to others where MRSA was detected only sporadically. Patient-to-patient transmission is considered the primary mechanism for the spread of this nosocomial pathogen [65]. While patient isolation can help reduce transmission, in practice, this is extremely difficult to implement, particularly in settings where entire wards or units are already colonized [66,67].

Members of the *Enterobacteriaceae* family, *Escherichia coli*, *Proteus* spp., and *Klebsiella* spp., as well as *Enterococcus* spp., were consistently isolated across nearly all studies. Their frequent detection, particularly in pelvic and perineal pressure ulcers, is likely related to proximity to the gastrointestinal tract and contamination with fecal flora [43,56,58]. This is especially relevant in patients with paraplegia, many of whom experience sphincter dysfunction, resulting in incontinence and increased risk of wound contamination and subsequent infection [68].

Streptococci were also commonly isolated (22.7% of all ulcers). While many streptococci are part of the normal human flora and may be interpreted as colonizers or contaminants of chronic wounds, certain species such as Group A (*S. pyogenes*) and Group B (*S. agalactiae*) exhibit sufficient virulence that their presence is almost invariably regarded as causative of infection [69]. In this context, when quantitative cultures are obtained from chronic wounds, microbial loads greater than 10⁵ CFU (colony-forming units) per gram of tissue are generally considered indicative of infection [68,69]. Robson and Heggers, however, identified β-hemolytic streptococci as unique among bacteria in their ability to cause clinically relevant infection even at levels significantly below this threshold, underscoring their exceptional pathogenic potential [70]. Historical observations reinforce this point: as early as 1918, Wright et al. had already noted that surgical wounds could not be successfully closed with sutures if hemolytic *Streptococcus pyogenes* was present [71]. Although the detection of such microorganisms frequently raises concern among clinicians, in pressure ulcers, their mere presence is of uncertain significance, as no definitive etiological link to infection can be established from cultures alone in these polymicrobial environments [72]. Moreover, microbial interactions can influence virulence expression and the clinical behavior of individual species, partly through a phenomenon known as quorum sensing - an intercellular signaling mechanism that allows bacteria to regulate pathogenicity once a critical population density is reached [73,74]. For example, Group A β-hemolytic streptococci have historically been associated with fulminant necrotizing fasciitis [75], yet such cases are uncommon in pressure ulcer-related-necrotizing infections, which are typically polymicrobial and present with less aggressive clinical features [43]. In our analysis of sampling methods, *Streptococcus* spp. were significantly more isolated in deep biopsies than in superficial swab samples. We hypothesize that this difference reflects the tendency to disregard streptococci as contaminants in swabs, in contrast with their recognition as pathogens in deep invasive specimens [13].

*Pseudomonas* spp., on the contrary, were more frequently associated with swab samples, likely reflecting their opportunistic ability to transiently colonize moist, exposed wound environments [59,76]. Clinically, *Pseudomonas aeruginosa* infection is often suspected based on its characteristic odor and green exudate [77,78], but culture remains essential for antimicrobial susceptibility testing, given its well-known multidrug resistance [39,76,78].

The analysis of anaerobic bacteria was particularly challenging. Nearly half of the included studies either did not perform anaerobic cultures, did not report them, or presented their results without differentiation, making them unsuitable for statistical analysis. Among studies that did provide data, anaerobes were most often isolated from necrotizing or complicated wounds where tissue hypoxia and devitalization create favorable conditions for their growth [30,43,59]. Examples include Sapico et al. [5] and Mizokami et al. [43] for necrotizing ulcers and Ehrenkranz et al. [33] and Brunel et al. [46] for draining or complicated wounds. In contrast, Brook et al. [34] reported a high prevalence without clear predisposing factors. Many authors suggest that anaerobes may represent a substantial proportion of microorganisms in chronic wounds, yet they often remain undetected due to methodological challenges in collection, transport, and culture. [5,59,79,80] Their presence has been associated with impaired wound healing and an increased risk of severe complications, such as gangrenous necrotizing infections and septicemia, with anaerobes frequently isolated from blood cultures in patients with pressure ulcer infections [14,42,43,59]. Anaerobes are characteristically found in polymicrobial settings [43,81], as they are commonly introduced together with other microorganisms - for example, through fecal contamination in perineal ulcers [5,47] - and are frequently dependent on aerobic species for survival and proliferation [80-82]. Clinically, acute anaerobic infection should always be suspected when a putrid odor is present, as this feature is considered uniquely characteristic of this group of pathogens [5,83]. This reliable association has provided the basis for recommending topical metronidazole gel to manage odor in putrid chronic wounds of various etiologies [84,85]. With regard to diagnostic sampling, in our analysis, neither *Bacteroides* spp. nor *Peptostreptococcus* spp. showed significant differences in detection between invasive and noninvasive collection techniques.

Another notable finding was the frequent reporting of *Corynebacterium* spp. and coagulase-negative *Staphylococcus*. These organisms, usually considered part of the normal skin microbiota, remain of uncertain pathogenic significance in pressure ulcer infection [55,86]. Indeed, approximately one-third of the studies appeared to exclude them from final reports, likely due to their common classification as contaminants in routine laboratory workflows [60]. Nevertheless, it should be emphasized that coagulase-negative *Staphylococcus* together with *Streptococcus* spp., *S. aureus,* and gram-negative bacilli are among the most frequent microorganisms implicated in surgical site infections in orthopedic procedures and therefore are frequently targeted by perioperative prophylactic antibiotic regimens in selected surgeries [87]. The pathogenic significance of these microorganisms should not be underestimated, especially in light of their ability to form biofilms, a mechanism that promotes their persistence and enhances the survival of neighboring bacteria [73,87,88].

Numerous studies have compared the diagnostic accuracy of sampling methods, with reported concordance between swab and biopsy cultures varying widely, from as low as 22% in the study by Tedeschi et al. [9] to as high as 93% in earlier work by Ehrenkranz et al. [33], with different reviews confirming substantial heterogeneity across settings [13,58]. Swabs can identify potential pathogens, but tissue biopsies are often regarded as more reliable for quantifying bacterial load and detecting deep tissue organisms, thereby helping to distinguish colonization from true infection [13,19]. Nevertheless, clinicians should bear in mind that cultures alone cannot confirm infection, which remains a clinical diagnosis [11,56]. An etiological role of a microorganism in infection or delayed healing can only be firmly established when it is present as a pure monomicrobial flora [72]; however, pressure ulcer cultures are inherently polymicrobial, and different ulcer sites often yield distinct microbial isolates [5,12,58]. Moreover, as Kingston and Seal have argued, all species associated with a microbial disease should be regarded as potentially synergistic rather than attributing causation to a single organism [89]. Such microbial synergy may amplify the overall pathogenic effect and thereby increase the severity of infection [72]. For this reason, sampling approaches should not be considered in terms of superiority or inferiority but rather as complementary techniques, each capable of capturing different aspects of the wound microbiome. Genetic sequencing studies of pressure ulcer samples have further demonstrated that a single ulcer can harbor hundreds of microbial species simultaneously [79,90], underscoring that conventional culture methods substantially underestimate microbial diversity [91,92]. Thus, while our findings provide important insights into predominant pathogens and the influence of sampling techniques, they must be interpreted with caution, recognizing that no single method captures the full picture and that results should always be integrated with clinical assessment [5,86].

Practical Implications of Antibiotic Treatment

As previously observed, pressure sores harbor a diverse and dynamic polymicrobial population that is neither evenly distributed nor stable over time [86]. Colonization often persists indefinitely, making prolonged antibiotic therapy unsustainable and an inefficient use of healthcare resources [60], an issue further compounded by rising antimicrobial resistance [7]. In this context, treatment strategies have shifted toward targeted antibiotic use, aiming both to preserve the efficacy of existing drugs and to reduce the spread of multidrug-resistant organisms [8,93]. Consequently, most guidelines recommend systemic antibiotics in pressure ulcers only when there is proven infection, such as cellulitis, fasciitis, osteomyelitis, bacteremia, or sepsis [1,69,94]. While we broadly support this position, our clinical experience as surgeons suggests that current recommendations may not fully address the reconstructive setting and that alternative strategies merit consideration.

Pressure ulcers are typically classified by the National Pressure Injury Advisory Panel (NPIAP) based on tissue depth. Stage I involves non-blanchable erythema of intact skin; stage II, partial-thickness skin loss with exposed dermis; stage III, full-thickness skin loss with visible subcutaneous fat but no bone, tendon, or muscle exposure; and stage IV, full-thickness tissue loss with exposed or palpable bone, tendon, or muscle. Unstageable ulcers have obscured wound bases due to slough or eschar, while pressure ulcers with suspected deep tissue injury present as persistent dark red or purple discoloration, indicating underlying deep tissue damage [1]. Advanced (stage III-IV) pressure ulcers, especially those in the pelvic region (such as ischial, sacral, or trochanteric), frequently require surgical intervention. [95]. These lesions have a healing rate as low as 18% and carry a recurrence rate of up to 77% when managed with nonsurgical approaches [96]. Radical excision of all ulcerated tissue and infected bone remains the cornerstone of surgical treatment [1], followed by reconstruction using fasciocutaneous or musculocutaneous flaps [95,97]. However, surgically treated pressure injuries, particularly in the pelvic region, are frequently associated with postoperative complications [98], with overall rates reported as high as 58% and wound dehiscence occurring in up to 49% of cases [1]. While multiple factors can contribute to wound breakdown, many authors identify surgical site infection as the final common pathway in most instances [99].

Several strategies have been proposed to reduce bacterial bioburden and improve surgical outcomes. The first and most critical step is thorough debridement of all nonviable tissue, including abnormal skin, granulation tissue, necrotic areas, sinus tracts, and slough, followed by superficial ostectomy of exposed bone [1,69,95] and meticulous cleaning and disinfection to disrupt and remove residual biofilm [69,100]. The second principle is elimination of dead space [69] through techniques such as deep flap anchoring [101,102], placement of surgical drains [69,95,103], and the use of antibiotic-impregnated surgical beads [104], thereby minimizing bacterial proliferation in accumulated exudate. The third principle involves creating a clean surgical environment that prevents contamination and promotes healing. In this context, sealing the suture line with topical skin adhesives (cyanoacrylate glue) may provide an additional barrier against external contamination and reduce the risk of superficial infection [105]. Other adjunctive measures such as fecal or urinary diversion, topical antiseptics and antibiotics, exudate-absorbing dressings, and the use of closed-incision negative-pressure wound therapy may also help reduce superficial contamination [8,69,99], though they are generally less effective in preventing bacterial growth within deeper tissues during the postoperative period [1]. Despite these strategies and standardized protocols, postoperative infection and wound dehiscence remain common [102,106]. In severe cases, infection may cause the reconstructed ulcer to fully reopen, resulting in an extended hospital stay, increased patient morbidity, and the need for prolonged antibiotic treatment [98], thereby exposing the patient to the risk of developing multidrug-resistant organisms [59]. This raises the question of whether some of these complications could have been avoided if the initial infection had been prevented.

Empirical regimens for pressure ulcers are typically recommended to provide coverage for gram-positive organisms (particularly MRSA and *Enterococcus *spp.), gram-negative bacteria (including *Pseudomonas* spp. and *Enterobacteriaceae*), and anaerobes, with constant consideration of local resistance patterns [11,57,80,107,108]. According to current guidelines and reviews, empiric antibiotic therapy is considered appropriate only in cases of life-threatening infection or sepsis, or while awaiting culture results from infected pressure ulcers, after which treatment should be adjusted to the sensitivity patterns of the isolated microorganisms [1,58,69,100]. In parallel, these same guidelines emphasize the polymicrobial and dynamic nature of pressure ulcers and discourage routine sampling because of the limited sensitivity and specificity of its methods [1,58,69,100]. As a result, targeted antibiotic therapy that relies on correct identification of the causative pathogen is often guided by uncertain or incomplete microbiological results and may simply not be sufficient in the perioperative scenario [9,11,48]. Focusing on a single pathogen in a polymicrobial environment risks selecting for resistant organisms that thrive in the absence of microbial competition [79,109,110] and may contribute to horizontal transfer of resistance genes, ironically fostering the emergence of multidrug-resistant organisms despite the opposite intent [4,59,82]. Moreover, this process takes time; from sample collection to isolation and susceptibility testing, several days or even weeks (in case of anaerobes) may pass. This delay can be critical, and in many clinical settings, such time is simply not available [9].

Therefore, a recommendation gap has arisen from the lack of context-specific evidence, as surgical practice continues to rely on publications that overlook reconstructive considerations [7,81]. Only a few surgeons have addressed this issue, advocating short perioperative courses of broad-spectrum antibiotics, albeit with differing regimens and strategies, and reporting variable complication rates [95,108,111-113]. However, to our knowledge, not a single controlled trial comparing complication rates in patients undergoing surgical treatment for pressure ulcers with and without perioperative broad-spectrum antibiotics has been published to date [7]. A short course of broad-spectrum antibiotics during the perioperative period (7-10 days) targeting the most frequently isolated pathogens, informed by local resistance patterns, could potentially prevent the need for prolonged postoperative antibiotics to treat complications and recurrences [35,53,111]. The rationale is to comprehensively address the full spectrum of likely pathogens at the time of surgery to reduce the risk of infection and wound breakdown [79,95,99]. This strategy may be compared to the multidrug treatment regimens used for *Helicobacter pylori* or *Mycobacterium tuberculosis*, in which combinations of antibiotics, selected with consideration of pathogen susceptibility, are employed to eradicate all potential strains and to prevent the emergence of additional resistance [114,115].

In our opinion, targeted antimicrobial therapy remains appropriate in specific scenarios, namely in cases of active infection, bacteremia, and select cases of osteomyelitis [1,69,94]. It is also important to emphasize that we do not advocate for definitive surgical coverage of pressure ulcers during acute infection or septicemia [69]. In this context, adjunctive biomarkers (particularly procalcitonin and C-reactive protein) may aid in the early detection of systemic or local infections and, in select cases, even assist in identifying osteomyelitis, thereby supporting surgical planning and ensuring that patients are adequately stabilized before reconstruction. Such biomarkers may also prove useful for assessing the efficacy of ongoing antibiotic therapy or detecting early postoperative complications [116,117]. Nevertheless, pressure ulcer-specific evidence remains limited, and we believe these biomarkers should not serve as primary decision-making tools for perioperative antibiotic management in this patient population.

Regarding osteomyelitis, both its diagnosis and the distinction between acute and chronic forms remain subjects of ongoing debate [16,46,118]. Contrary to the common perception that exposed bone must invariably indicate osteomyelitis, several case series based on bone biopsies have shown that only a minority of such patients exhibit inflammatory changes within bone tissue, while most demonstrate only pressure-related alterations [16,31,119]. Moreover, even in diagnosed chronic osteomyelitis, bacteria are often not identifiable histologically, reinforcing the view that these lesions may in some cases be adequately managed with surgical resection alone, without the need for prolonged antibiotic therapy [118]. Nevertheless, despite the fact that only a subset of exposed bones demonstrates true bacterial osteomyelitis, a widely held opinion is that all chronic wounds with exposed bone, such as stage IV pressure ulcers, should raise suspicion for underlying osteomyelitis, which ought to be actively investigated and, if confirmed, managed with bone resection, culture, and extended courses of systemic antibiotics [104,120]. Paradoxically, certain clinical series have not demonstrated higher postoperative complication rates among pressure ulcer patients with confirmed osteomyelitis, and some even described lower rates in this subgroup [15,40,52,54], a finding that may be explained by more aggressive surgical debridement or by the obligatory prolonged antibiotic regimens to which this subgroup is subjected, in contrast to patients with pressure ulcers without a formal osteomyelitis diagnosis. With regard to treatment duration, accumulating evidence indicates that shorter therapeutic regimens of one to four weeks of targeted antibiotics may be adequate, provided that complete resection of the affected bone is performed in combination with appropriate soft tissue coverage [53,81,119]. Taken together, these findings suggest that radical bone resection represents the cornerstone of effective management, whereas the role of postoperative antibiotic therapy, particularly regarding its optimal duration, remains uncertain.

In no circumstance should antibiotic therapy, whether targeted or empirical, be employed in isolation, without simultaneously addressing factors such as dead space, exudate accumulation, necrotic tissue, and biofilm, all of which can severely compromise its penetration and therapeutic effectiveness [69,80,121,122]. Recent research on biofilm dynamics has highlighted its critical role in delayed wound healing and antibiotic resistance, functioning both as a physical barrier and as a persistent reservoir of senescent bacteria [42,82,123,124].

Postoperative care must also include appropriate dressing management, patient positioning, specialized mattresses, nutritional optimization, physical therapy, and social support [1,69,105]. The treatment of pressure ulcers should focus on the patient as a whole, not just the wound, with antibiotic therapy representing only one component of a comprehensive, multidisciplinary approach [8,95].

Limitations

Limitations for comparability arose from the inclusion of very different populations, treated in diverse care settings and countries over the past 50 years. Studies used distinct sampling and culture methods, reported results in different ways, and applied varying criteria for contaminants, duplicates, and anaerobic cultures. These methodological and reporting differences make direct comparison difficult, yet it is noteworthy that despite such variability, the overall picture remains remarkably consistent: Pressure ulcers are polymicrobial wounds in which a core group of around 10 bacterial species tend to predominate, albeit in slightly different proportions.

Although only 11 of the 28 studies sampled patients in the reconstructive surgical context, they accounted for about two-thirds of the total population (≈66.8%). Approximately 65% of patients were male, and more than 60% presented with spinal cord injury, which is more common in men, due to the higher frequency of traffic and work-related accidents [125]. Only four studies included nonhospitalized individuals, and just one involved pediatric patients, representing less than 9% and 4% of the overall sample, respectively. As a result, the evidence is heavily weighted toward hospitalized adult populations with spinal cord injury in the surgical context, while community-based cases remain underrepresented. The ulcers analyzed were also predominantly stage III and IV, which constitute less than 10% of all pressure injuries [126] but are the only lesions with a formal indication for surgical treatment [96]. Consequently, while these findings have limited generalizability to early-stage or community-managed pressure injuries, they strengthen the discussion of how this microbiological profile should inform reconstructive protocols currently in use, particularly with regard to bacterial identification and antimicrobial treatment.

## Conclusions

This review highlights the complex and heterogeneous microbiology of pressure ulcers and the considerable variability in reported findings across studies. Although* Staphylococcus aureus* and *Enterobacteriaceae* remain the most frequently isolated pathogens, pressure ulcers typically harbor a diverse and dynamic microbial community shaped by sampling technique, culture conditions, and patient-specific factors. Current evidence indicates that a comprehensive microbiological characterization of pressure ulcers is still lacking, and our understanding of microbial interactions and their adaptation to the wound environment remains limited.

Despite decades of clinical experience, complication rates following surgical treatment of pressure ulcers remain unacceptably high, with only modest improvements achieved over the past 50 years. Current guidelines recommend restricting antibiotic use to confirmed infections, but clinical experience suggests that this approach may be insufficient in the surgical context. The potential benefits of short perioperative courses of broad-spectrum antibiotics directed at the most commonly isolated pathogens in pressure ulcers warrant further investigation, and prospective controlled trials are urgently needed. Although antibiotic therapy is important, it should be regarded as only one component of a broader multidisciplinary strategy that also addresses patient comorbidities, nutritional and immune status, care environment, local resistance patterns, and wound-specific factors such as necrotic tissue, biofilm, dead space, and osteomyelitis.

## Figures and Tables

**Figure 7:**
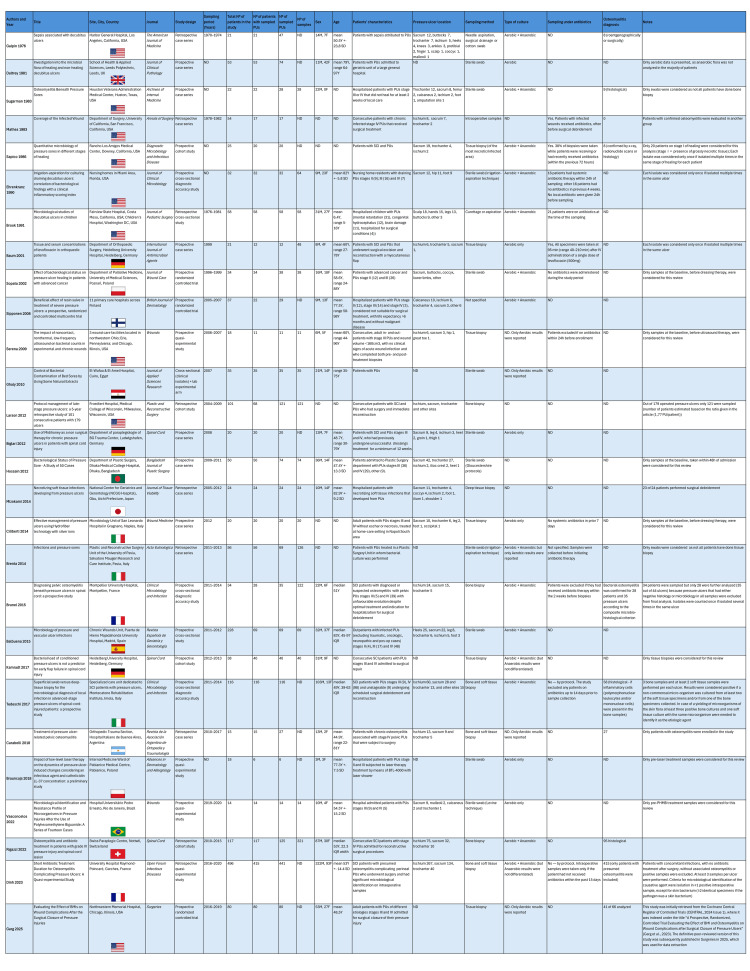
Detailed characteristics of the included studies, patients, and pressure ulcers This figure reproduces and expands on the information presented in Table 2 of the main manuscript, providing additional methodological and contextual details. Nº = number, M = male, F = female, Y = years, PU = pressure ulcer, ND = no data, SD = standard deviation, IQR = interquartile range, USA = United States of America, UK = United Kingdom, SCI = spinal cord injury, BMI = body mass index.
