# Correction: Microbiology of Pressure Ulcers: A Systematic Review

**DOI:** 10.7759/cureus.c384

**Published:** 2025-12-19

**Authors:** Dmitry Shelepenko, Beatriz Jardim, Luís M Almeida, José Miguel Azevedo, Inês Catalão, Miguel Sítima, Gonçalo Tomé

**Affiliations:** 1 Department of Plastic and Reconstructive Surgery and Burns Unit, Coimbra Local Health Unit, Coimbra, PRT; 2 Department of Public Health, Local Health Unit of the Leiria Region, Leiria, PRT; 3 Department of Plastic and Reconstructive Surgery, CUF Santarém, Santarém, PRT

This article has been corrected to convert tables 3, 4, and 5 to figures in order to preserve the formatting and color present in the original tables. The remaining tables and figures have been re-ordered accordingly.

